# Editorial: Molecular targets for the treatment of metastatic colorectal cancer

**DOI:** 10.3389/fonc.2023.1341594

**Published:** 2023-12-12

**Authors:** Alessandro Passardi, David Gibbons

**Affiliations:** ^1^ Department of Medical Oncology, IRCCS Istituto Romagnolo per lo Studio dei Tumori (IRST) “Dino Amadori”, Meldola, Italy; ^2^ Department of Pathology, St Vincent’s University Hospital, Dublin, Ireland; ^3^ School of Medicine and Medical Sciences, University College Dublin, Dublin, Ireland

**Keywords:** colorectal cancer, molecular targets, metastatic colon cancer, angiogenesis, EGFR inhibitiors, immunocheck point inhibitors, refractory colorectal cancer, BRAF V600E

Colorectal cancer (CRC) accounts for approximately 10% of all cancer cases and represents the third most common cancer worldwide. Most importantly, it is the second leading cause of cancer-related deaths worldwide. The disease is often diagnosed at an advanced stage, when treatment options are limited (1, 2).

From the 1990s on, fluorouracil-based chemotherapy was used to treat metastatic CRC (mCRC), improving overall survival (OS) to 14 months. Later, the combination regimens with oxaliplatin (FOLFOX) or irinotecan (FOLFIRI) prolonged the OS to about 20 months (3). From the early 2000s targeted drugs, like anti-epidermal growth factor receptor [EGFR] or anti-vascular endothelial growth factor [VEGF] antibodies, have entered clinical practice, significantly increasing patients’ OS to approximately 36 months (4).

Antiangiogenic agents, such as Bevacizumab and Aflibercept, are widely used in combination with first and second line chemotherapy for mCRC (4). Despite several years of translational research in this field, no validated predictive markers have been found to select patients more likely to benefit from these agents. A multidisciplinary group from University Hospital of Parma, Italy, performed an interesting trial to investigate the role of the Notch intracellular domain (NICD) and its ligand Jagged-1 expression, as well as radiomics in the prediction of the efficacy of bevacizumab in treatment-naive metastatic CRC patients. Study results, presented in this Research Topic, suggested that high NICD and Jaged-1 expression levels were associated with early disease progression (Negri et al.). Moreover, the integration of quantitative information combined with clinical and histologic characteristics helped predict patient outcomes. This seems to be a promising field of research, which needs validation in larger cohorts of patients. Vanucizumab (RO5520985), a humanised immunoglobulin G-1-like bispecific monoclonal antibody targeting both VEGF-A and Angiopoietin-2, has been recently evaluated In the phase II McCAVE trial in combination with FOLFOX first line chemotherapy, showing similar efficacy in terms of PFS and OS compared to bevacizumab. In the attempt to find new predictors for outcome related to the anti-angiogenic treatment, Ferreira et al. explored the potential predictive and prognostic role of baseline tissue and plasma levels of Angiopoietin-2 in a subgroup of patients enrolled into the Mc Cave trial. Overall, low tissue baseline levels of Angiopoietin-2 were associated with longer PFS. Moreover, patients with KRAS wild-type mCRC and high levels of Angiopoietin-2 had higher PFS when treated with vanucizumab with respect to bevacizumab.

EGFR plays a key role in colorectal tumorigenesis, and acts to activate several intracellular signalling pathways, such as the RAS-RAF-MAP kinase and the PI3K-PTEN-Akt pathway, thus favouring cell proliferation, migration and differentiation. Anti-EGFR antibodies, cetuximab and panitumumab, are widely used for mCRC patients, in particular in combination with first line chemotherapy in patients with left sided RAS/BRAF WT tumors (4). In this subgroup of patients, in addition to significantly increasing OS, these combination therapies may allow conversion of unresectable to resectable liver metastases, thus expanding the possibilities of cure in mCRC. The LM02 trial, presented in this Research Topic, evaluated FOLFIRI plus panitumumab regimen as perioperative therapy in untreated RAS WT mCRC patients with liver limited disease [Piringer et al.]. Among the 36 patients included, 91.4% completed the preoperative therapy. The objective response rate and R0 resection rate were 65.7% and 82.7%, respectively. Noteworthy, The OS rates at 12 and 24 months were 85.6% and 73.3%, respectively. Unfortunately, despite great efforts to select patients addicted to anti-EGFR blockade, treatment efficacy suffers from either innate or acquired mechanisms of resistance, largely driven by RAS or BRAF mutations. Liquid biopsy analysis with the detection of such and other mutations might help monitor tumour spatial and temporal heterogeneity and predict resistance to anti EGFR agents. It has recently been recommended to select patients for the use of anti-EGFR drugs beyond progression or as rechallenge strategy. The phase II CAPRI 2 GOIM trial, a proposal, presented in this Research Topic by Martini et al., is a clinical trial designed to follow RAS/BRAF wild type mCRC cases, as determined on initial FFPE diagnostic tissue, through three lines of therapy to include FOLFIRI, Cetuximab, Folfox and bevacizumab in various combinations, depending on dynamic mutation changes with time, as measured by Liquid Biopsy analyses, after each line of treatment. Endpoints will include Response rate (RR), PFS and OS.

More recent acquisitions include the use of immunocheckpoint inhibitors (ICIs), i.e. Pembrolizumab or Nivolumab/Ipilimumab combination, in mCRC patients with Microsatellite instability or deficient Mismatch Repair. However, approximately 95% of mCRC are microsatellite-stable/mismatch-repair-proficient, and this condition involves resistance to ICIs (5). The molecular mechanism of ICI resistance is largely unknown and clinical research is addressing the complex issue of transforming tumors from the immune “cold” state to the immune “hot” state. Insufficient CD8+ T cell infiltration or loss of CD8+ T cell function might restrict the efficiency of immunotherapy in CRC. In this context, Tan et al. found that matrix remodelling associated protein 8 (MXRA8) was over expressed in CRC, significantly affecting tumor malignancy, metastasis and recurrence. Moreover, MXRA8 seemed to correlate with CRC immunity, reflecting an abnormal immune status, characterized by less infiltration or dysfunction of CD8+ T cells. Therefore, MXRA8 might be implemented as a potential immunotherapeutic and prognostic biomarker for CRC.

An increasing number of patients with mCRC are able to receive 3 or more lines of therapy and in recent years the therapeutic armamentarium in this setting has significantly expanded. In particular, regorafenib, an oral multikinase inhibitor, and trifluridine/tipiracil, an oral fluoropyrimidine, represent the standard treatment options for chemorefractory mCRC patients. In the Correct and Recourse trials, Regorafenib and trifluridine/tipiracil showed a significant OS improvement in comparison to best supportive care [HR 0.77 (IC 95% 0.64-0.94), p 0.0052; HR 0.66 (IC 95% 0.56-0.78), p<0.001, respectively] (6, 7). Another option in this setting includes (even if with evidence from only phase 2 trials), the rechallenge with EGFR inhibitors in RAS/BRAF WT tumors. Salvatore et al. present here a retrospective trial to assess the efficacy, according to tumor site, of the different treatment regimens (anti-EGFR-based therapy versus regorafenib or trifluridine/tipiracil) in refractory RAS/BRAF wt mCRC patients. They found a significant benefit in terms of OS in favour of anti-EGFR therapy in the left sided tumor group, whereas no differences were observed in the right sided tumor group. These results suggest an opportunity, to be confirmed in randomized trials, to select left side tumors for antiEGFR treatment in later lines. Therapeutic options in refractory disease will further increase in the coming years, thanks to the introduction of Fruquintinib and the combination of trifluridine/tipiracil with bevacizumab, the efficacy of which has recently been demonstrated in phase III studies. Furthermore, combination therapies of these drugs are underway, especially with ICIs, which could take a further step forward in the fight against CRC. Xue et al. carried out a meta-analysis of 22 studies including 1,866 patients with refractory mCRC treated with targeted therapies as third or later line of treatment. The pooled ORRs for VEGF and EGFR inhibitors were 4% and 19%, respectively. More favourable objective response and disease control rates were reported for patients treated with combined treatments with respect to monotherapy. Larger well-designed clinical trials are expected to better analyze efficacy and safety of VEGF and EGFR inhibitors, as well as combined strategies (in particular with ICIs), in the treatment of refractory mCRC.

Another key molecular target in CRC is BRAF. In particular, BRAF^V600E^ mutations are present in about 12% of mCRC and are associated with right sidedness, poor differentiation, and mucinous-type tumors, but above all with a poor disease prognosis and a poor response to standard therapies. The European Medicines Agency (EMA) has recently approved doublet therapy with encorafenib, a kinase inhibitor of BRAF, and cetuximab as second or third line treatment for BRAF^V600E^ mCRC, according to the results of the phase III Beacon trial. This targeted treatment is under investigation in combination with both first line chemotherapy and ICIs in MSI mCRC patients. Moreover, other BRAF inhibitors, such as vemurafenib and dabrafenib are being evaluated in clinical trials. Piringer et al. report, in this Research Topic a patient case with an impressive therapeutic result (i.e. a complete remission still persisting after several years) in a 52-year-old woman with advanced BRAF^V600E^ mutated, MSS mCRC, treated with dabrafenib, trametinib, and cetuximab as later-line therapy.

There is a growing need for clinical and preclinical research aimed at identifying new targets for the selective treatment of mCRC. Among the new tumor targets under development, abnormal gene splicing is emerging as a process able to promote tumor cell proliferation and invasion, resistance to apoptosis and probably resistance or sensitivity to chemotherapy. Numerous splicing isoforms have been identified, that are appropriate candidates for targeted treatment, even in mCRC (Zheng et al.). Zhou et al. deeply investigated the role of Anoikis and epithelial-mesenchymal transition (EMT) in the occurrence of distant metastasis of CRC. In particular, they focused on the understanding of their crosstalk and the identification of key genes. Besides the prognostic role, these findings could help in developing novel therapeutic targets for patients with mCRC. A further new frontier in the selective treatment of solid tumors, including mCRC, is nanomedicine. Nanoparticles are able to maximize treatment efficacy, by directly targeting cancer cells and regulating drug release [Jain and Bhattacharya]. The review by Jain and Bhattacharya carefully describes the nanomaterials that can be employed, as well as the preparation techniques and targeting mechanisms. Even though this field of research seems promising, more data from preclinical and clinical studies are eagerly awaited to bring this technology to the market.

## Author contributions

AP: Writing – original draft, Writing – review & editing. DG: Writing – original draft, Writing – review & editing.
